# Phonon Scattering and Suppression of Bipolar Effect in MgO/VO_2_ Nanoparticle Dispersed p-Type Bi_0.5_Sb_1.5_Te_3_ Composites

**DOI:** 10.3390/ma14102506

**Published:** 2021-05-12

**Authors:** Song Yi Back, Jae Hyun Yun, Hyunyong Cho, Gareoung Kim, Jong-Soo Rhyee

**Affiliations:** 1 Department of Applied Physics, Integrated Education Institute for Frontier Science and Technology (BK21 Four) and Institute of Natural Sciences, Kyung Hee University, Yongin 17104, Korea; song2back@gmail.com (S.Y.B.); ataxtr@hanmail.net (J.H.Y.); gprhf1@naver.com (H.C.); 2 Energy Materials Laboratory, Toyota Technological Institute, Nagoya 468-8511, Japan; noah04@naver.com

**Keywords:** thermoelectric, bismuth telluride, oxide nanoparticle composite, phonon scattering

## Abstract

Bismuth-Telluride-based compounds are unique materials for thermoelectric cooling applications. Because Bi_2_Te_3_ is a narrow gap semiconductor, the bipolar diffusion effect is a critical issue to enhance thermoelectric performance. Here, we report the significant reduction of thermal conductivity by decreasing lattice and bipolar thermal conductivity in extrinsic phase mixing of MgO and VO_2_ nanoparticles in Bi_0.5_Sb_1.5_Te_3_ (BST) bulk matrix. When we separate the thermal conductivity by electronic $\kappa_{el}$, lattice $\kappa_{lat}$, and bipolar $\kappa_{bi}$ thermal conductivities, all the contributions in thermal conductivities are decreased with increasing the concentration of oxide particle distribution, indicating the effective phonon scattering with an asymmetric scattering of carriers. The reduction of thermal conductivity affects the improvement of the *ZT* values. Even though significant carrier filtering effect is not observed in the oxide bulk composites due to micro-meter size agglomeration of particles, the interface between oxide and bulk matrix scatters carriers giving rise to the increase of the Seebeck coefficient and electrical resistivity. Therefore, we suggest the extrinsic phase mixing of nanoparticles decreases lattice and bipolar thermal conductivity, resulting in the enhancement of thermoelectric performance over a wide temperature range.

## 1. Introduction

Thermoelectric (TE) materials enable the direct conversion of waste heat into electricity and vice versa, and are applied to environmentally friendly energy harvesting. The TE efficiency is defined by the dimensionless figure-of-merit, $ZT=S^{2}T/\left(\kappa \rho \right)$, where S, T, ρ, and κ are the Seebeck coefficient, absolute temperature, electrical resistivity, and thermal conductivity, respectively.

A high TE performance is required for a high power-factor, $PF=S^{2}/\rho$, and a lower thermal conductivity. The Seebeck coefficient’s improvement can enhance the PF through the band engineering [1,2,3] and carrier filtering effect [4,5,6]. The nano-structuring [7,8] and secondary phase dispersion [9,10] can induce pronounced phonon scattering, which results in the reduction of thermal conductivity. The anharmonic lattice vibration leads to the intrinsic low lattice thermal conductivity [11,12]. There have been investigations on the high power-factor and low thermal conductivity through the Peierls distortion [13,14] and selective charge Anderson localization [15]. 

The Bi–Te-based alloys are unique TE materials near room temperature. The Bi_2_Te_3_ alloys have a narrow gap semiconductor, and the bandgap is ~0.13 eV [16], which gives rise to bipolar conduction in high temperatures. The bipolar diffusion effect deteriorates TE performance due to electron-hole compensation of the Seebeck coefficient. Therefore, the suppression of bipolar diffusion effect is a critical issue in Bi–Te-based thermoelectric materials. Doping or co-doping can reduce the thermal conductivity by inducing phonon scattering [17,18,19,20]. The Te-embedded Bi_2_Te_3_ thin films show a considerable reduction of lattice and bipolar thermal conductivity due to the Te–Bi_2_Te_3_ heterojunctions [21]. The nano-oxide particles are adopted in BST materials as a scattering center of charge carriers and phonons, reducing thermal conductivity [22,23,24]. The composite of BST/oxide materials has the interface between oxide elements and BST matrix by the dispersion of oxide particles. Even though the power factors are decreased, the ZT values are enhanced due to the strong thermal conductivity reduction [23,24]. 

Here, we investigated the thermoelectric properties of MgO/VO_2_ Bi_0.5_Sb_1.5_Te_3_ composites. The MgO and VO_2_ phases are stable during high temperature sintering process so that they remain as secondary phases in the BST matrix. It has been also reported that nano-particle dispersion in BST matrix significantly enhances thermoelectric performances [25,26,27,28]. The significant reduction of thermal conductivity for oxide composites is observed. The two-band model for thermal conductivity analysis confirmed that the electronic thermal conductivity is greatly reduced and the bipolar and lattice thermal conductivity are also decreased by MgO and VO_2_. The MgO and VO_2_ play a role as a scattering center of charge carriers. Therefore, the electrical resistivity increases and the electronic thermal conductivity decreases. The ZT of 5 mol.% VO_2_/BST composite is enhanced over the entire measured temperature range due to the reduction of thermal conductivity while the powder factor remains similar to the value of BST.

## 2. Materials and Methods 

### 2.1. Synthesis

The MgO/VO_2_ Bi_0.5_Sb_1.5_Te_3_ composites were synthesized by extrinsic phase mixing with oxide nanoparticles. Pristine Bi_0.5_Sb_1.5_Te_3_ (BST) was synthesized by direct melting. Stoichiometric elements of high purity Bi, Sb, and Te (>99.99%, Alpha Aesar, Ward Hill, MA, USA) were sealed into vacuum quartz tubes, melted at 1023 K for 10 h, and then quenched in cold water. The ingots were hand ground into fine powders. The nanopowders of MgO and VO_2_ (>99%, 100 nm, Alpha Aesar, Ward Hill, MA, USA) were mixed with 15 g of BST powder in hexane 20 mL for 12 h under 80 RPM. The compositions are Bi_0.5_Sb_1.5_Te_3_ + (5 and 10) mol.% of X, where X = MgO and VO_2_. The powders obtained after drying hexane were sintered at 623K (below the melting point of Te, 723 K) for 1 h under a uniaxial pressure of 50 MPa using a vacuum hot-press (Y&I Tech, Paju, Korea). All samples were cut and characterized in a direction parallel to the hot-press direction. 

### 2.2. Characterization

The powder X-ray diffraction (XRD) of sintered samples at room temperature was conducted by Cu Kα radiation (D8 Advance, Bruker, Billerica, MA, USA). The microstructure analysis was characterized using a high-resolution field emission scanning electron microscope (HR FE-SEM, MERLIN, Carl Zeiss, Baden-Württemberg, Germany) with the energy dispersive spectroscopy (EDS) mapping. The temperature-dependent electrical resistivity and Seebeck coefficient were measured under helium atmosphere using the commercial thermoelectric measurement system (ZEM-3, ULVAL-RIKO, Osaka, Japan). The Hall carrier concentration *n_H_* and carrier mobility *μ_H_* were obtained by using the relation $n_{H}=-1/\left(eR_{H}\right)$ and $\mu_{H}=1/\left(\rho en_{H}\right)$ where $R_{H}=\rho_{xy}/H$ is the Hall coefficient, and $\rho_{xy}$ is the Hall resistivity. The Hall measurement was performed by a physical property measurement system (PPMS, Dynalcool-14T, Quantum Design, San Diego, CA, USA) using a four-probe method. The thermal conductivity was calculated from the relation $\kappa =C_{p}d\lambda$, where $C_{p}$, d, λ are the specific heat, sample density, and thermal diffusivity. The thermal diffusivity was measured by a laser flash method (LFA-457, NETZSCH, Selb, Germany), and the heat capacity was obtained from PPMS.

## 3. Results and Discussion

**Figure 1:**
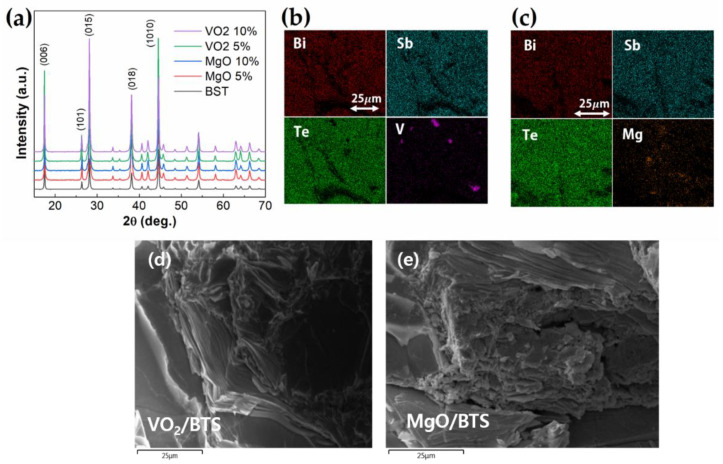
(**a**) X-ray diffraction patterns of MgO/VO_2_ BST composites; (**b**,**c**) are the elemental mapping images by energy dispersive X-ray spectroscopy (EDX) of the VO_2_ and MgO composite; (**d**,**e**) are the scanning electron microscope (SEM) images of the VO_2_ and MgO composite.

The X-ray diffraction (XRD) patterns for MgO/VO_2_ Bi_0.5_Sb_1.5_Te_3_ composites are shown in Figure 1a. All of the patterns show a single phase and could be indexed to the rhombohedral structure of Bi_2_Te_3_. All samples’ XRD patterns show no observable MgO and VO_2_ phases since a small amount of MgO and VO_2_ could not be detected in XRD. We calculated the average grain size d from the XRD patterns using Equation (1):

(1) $$B=\frac{0.9\;\lambda }{dcos\theta }$$

where B is the full width at half the maximum of the broadened diffraction line, d is a diameter of the crystallites, λ is the X-ray wavelength, 1.5406 A, and θ is the Bragg diffraction angle, respectively. The internal lattice strain was calculated by the Williamson-Hall equation [29]

(2) $$\frac{\beta \;cos\theta }{\lambda }=\frac{1}{d}+4\varepsilon \frac{sin\theta }{\lambda }$$

where β and ε are the integral breadth of the diffraction peak and the internal lattice strain, respectively. The obtained ε values are listed in Table 1. The lattice strains of oxide composite compounds are higher compared with that of pristine BST. It implies that the oxide particles generate the lattice strain, which can scatter the phonons.

The dispersion of oxide particles is investigated by the energy-dispersive spectroscopy (EDS) elemental map analysis for the samples for MgO 10 mol.%/BST composite (Figure 1b) and VO_2_ 10 mol.%/BST composite (Figure 1c). While Bi, Sb, and Te are homogeneous in compounds, V and Mg are distributed randomly, which shows the phase separation of VO_2_ and MgO in BST matrix. Figure 1d,e shows the scanning electron microscope (SEM) images of 10 mol.% dispersed VO_2_/BST and MgO/BST composites. Because the Bi_2_Te_3_-based compounds have layered crystal structure with van der Waals bonding layer along the c-axis, it shows the stacking along the c-axis, implying the anisotropic thermoelectric properties. It is generally known that the electronic transport properties are better along the in-plane direction rather than those of the out-of-plane direction, while the thermal conductivity along the out-of-plane direction is lower than that of the in-plane direction. In many cases, the thermoelectric performance along the out-of-plane direction is higher than that of the in-plane direction due to lower thermal conductivity, so we measured thermoelectric properties along the out-of-plane direction.

**Figure 2:**
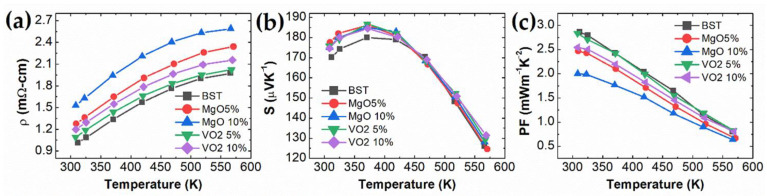
Temperature-dependent (**a**) electrical resistivity ρ(T); (**b**) Seebeck coefficient S(T); (**c**) power factor $S^{2}/\rho$ of MgO and VO_2_ BST composites.

The temperature-dependent electrical resistivity *ρ*, Seebeck coefficient *S*, and power factor PF =$\;S^{2}/\rho$ are shown in Figure 2. The electrical resistivity increases continuously with increasing temperature, indicating a metallic or degenerated semiconducting behavior. The Seebeck coefficient of all samples shows the positive value, consistent with the positive Hall coefficients, showing carriers’ p-type conduction. The electrical resistivity of MgO/VO_2_ Bi_0.5_Sb_1.5_Te_3_ composites increases with increasing oxide concentration such that the resistivity value of 10 mol.% is larger than the value of 5 mol.% in both MgO and VO_2_ cases. The Seebeck coefficient of MgO/VO_2_ Bi_0.5_Sb_1.5_Te_3_ composites slightly increased from room temperature to 425K, which is a minor change compared to the significant change in electrical resistivity. 

The MgO and VO_2_ dispersion in BST matrix can scatter charge carriers. We measured the Hall resistivity and estimated transport properties such as the Hall carrier concentration $n_{H}$ and Hall carrier mobility $\mu_{H}$ under 1T, which are listed in Table 1. The Hall carrier concentration of the MgO composite samples is systematically decreased with increasing oxide concentration, while those of VO_2_ dispersion are less sensitive with the oxide concentration in the matrix. On the other hand, Hall mobilities are systematically decreased with increasing oxide concentration in both MgO and VO_2_ dispersion composites. For example, the carrier mobility of BST is 189 ${\mathrm{cm}}^{2}\cdot V^{-1\;}\cdot s^{-1}$ and the values of oxide composite are decreased (161 ${\mathrm{cm}}^{2}\cdot \;V^{-1\;}\cdot s^{-1}$) for MgO 5 mol.% BST composite, 146 ${\mathrm{cm}}^{2\;}\cdot V^{-1\;}\cdot s^{-1}$ for MgO 10 mol.% composite, 173 ${\mathrm{cm}}^{2\;}\cdot V^{-1\;}\cdot s^{-1}$ for VO_2_ 5 mol.% composite, and 186 ${\mathrm{cm}}^{2\;}\cdot V^{-1\;}\cdot s^{-1}$ for VO_2_ 10 mol.% composite. The carrier mobility decrease is due to the carrier’s scattering near the grain boundary between the matrix and extrinsic micro-particles. Because the MgO and VO_2_ were not participating as doping in BST, the carrier concentration is less sensitive than Hall mobility. 

The effective masses of the MgO/VO_2_ BST composites are obtained using the single parabolic Pisarenko relation and listed in Table 1:

(3) $$S=\frac{8\pi^{2}k_{B}{}^{2}}{3eh^{2}}m^{*}T{\left(\frac{\pi }{3n}\right)}^{2/3}$$

where $k_{B}$, h, e, $m^{*}$, T, and n are the Boltzmann constant, Plank constant, elementary charge, effective mass of carrier, absolute temperature, and carrier concentration, respectively. Because there is no significant change of Seebeck coefficient and carrier concentration, the effective mass of oxide composites is close to BST. Because the oxide composites scatter carriers near the grain boundary, the electrical resistivity gradually increases with increasing MgO/VO_2_ concentration. BST/MgO 10%’s resistivity value is 1.5 times higher than that of pristine BST near room temperature. The composites’ power factors are decreased due to the increase in electrical resistivity and the less sensitive Seebeck coefficient with oxide dispersion. The decrease of power factor in the MgO/BST composite is more significant than that of the VO_2_/BST composite. The significant enhancement of carrier scattering in MgO/BST composite leads to the decrease of Hall mobility and increase of electrical resistivity, resulting in the decrease of power factor.

**Figure 3:**
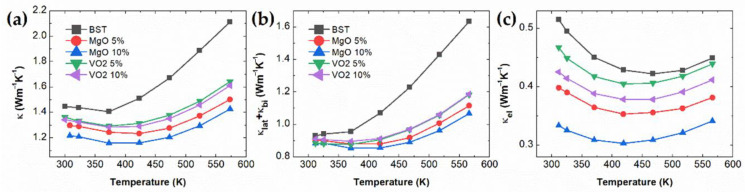
Temperature-dependent (**a**) total thermal conductivity κ; (**b**) lattice and bipolar thermal conductivity $\kappa_{L}+\kappa_{bi}$; (**c**) electronic thermal conductivity $\kappa_{el}$ of MgO and VO_2_ BST composites.

The temperature-dependent total thermal conductivity κ, calculated lattice $\kappa_{lat}$ and bipolar thermal conductivity $\kappa_{bi}$, and electronic thermal conductivity $\kappa_{el}$ are presented in Figure 3. The κ values are greatly reduced by introducing MgO and VO_2_ in the BST matrix, as shown in Figure 3a. The reduction of κ(T) of the MgO/BST composite is the most significant, mainly from high electrical resistivity. The lattice thermal conductivity can be obtained by subtracting the electronic thermal conductivity from the total thermal conductivity. According to Wiedemann-Franz’s law, the electronic thermal conductivity is given by $\kappa_{e}=L_{0}T\sigma$, where $L_{0}$ is the Lorenz number. To determine the Lorenz number for semiconductors with a single parabolic band model, the following Fermi integral formalism in Equation (4) is used:

(4) $$F_{n}\left(\eta \right)=\int_{0}^{\infty }\frac{x^{n}}{1+e^{x-\eta }}dx$$

where $F_{n}\left(\eta \right)$ is the nth order Fermi integral and $\eta =E_{F}/k_{B}T$ is the reduced chemical potential energy.

(5) $$\mathrm{The}\;S\left(T\right)\;\mathrm{is}\;\mathrm{calculated}\;\mathrm{by}\;\mathrm{Equation}\;(5):S=\pm \frac{k_{B}}{e}\left\{\frac{\left(r+\frac{5}{2}\right)F_{r+\frac{3}{2}}\left(\eta \right)}{\left(r+\frac{3}{2}\right)F_{r+\frac{1}{2}}\left(\eta \right)}-\eta \right\}$$

The temperature-dependent Lorenz number L(T) is given by Equation (6):

(6) $$L={\left(\frac{k_{B}}{e}\right)}^{2}\left\{\frac{\left(r+\frac{7}{2}\right)F_{r+\frac{5}{2}}\left(\eta \right)}{\left(r+\frac{3}{2}\right)F_{r+\frac{1}{2}}\left(\eta \right)}-{\left[\frac{\left(r+\frac{5}{2}\right)F_{r+\frac{3}{2}}\left(\eta \right)}{\left(r+\frac{3}{2}\right)F_{r+\frac{1}{2}}\left(\eta \right)}\right]}^{2}\right\}$$

where the scattering parameter r=−1/2 when the dominant scattering is acoustic phonon. The estimated Fermi energy $E_{F}$ on the compounds are 81.4 meV (Bi_0.5_Sb_1.5_Te_3_), 48.7 meV (BST/MgO 5%), 66.8 meV (BST/MgO 10%), 68.6 meV (BST/VO_2_ 5%), and 67.0 meV (BST/VO_2_ 10%), respectively. 

Bi_2_Te_3_-based materials are known as narrow-gap semiconductors, and the bandgap is about 0.13 eV [16]. The compounds give rise to bipolar conduction at high temperatures, which leads to the poor ZT value due to the increase in thermal conductivity. The thermal conductivity can be separated into three components:

(7) $$\kappa =\kappa_{el}+\kappa_{bi}+\kappa_{lat}$$

The bipolar and electronic thermal conductivities are calculated based on the two-band model with the coexistence of electron and hole, using the Boltzmann transport equation. The following equations can describe the thermoelectric properties in the two-band model:

(8) $$S_{tot}=\frac{S_{e}\sigma_{e}+S_{h}\sigma_{h}}{\sigma_{e}+\sigma_{h}}$$

(9) $$\sigma_{tot}=\sigma_{e}+\sigma_{h}$$

(10) $$\kappa_{el}=L_{elec}\sigma_{tot}^{exp}T$$

(11) $$\kappa_{bi}=L_{bi}\sigma_{tot}^{exp}T$$

where $L_{elec}=\frac{L_{e}\sigma_{e}+L_{h}\sigma_{h}}{\sigma_{e}+\sigma_{h}}$ is the electronic Lorenz number, $L_{bi}=\sigma_{e}\sigma_{h}{\left(\frac{S_{h}-S_{e}}{\sigma_{tot}}\right)}^{2}$ is the Lorenz number contributed from the bipolar transport, and $\sigma_{tot}^{exp}$ is the experimental total conductivity, respectively. When we calculated the bipolar thermal conductivity from the above equations, we found a reduction of bipolar thermal conductivity in the oxide composites, but the lattice thermal conductivity showed a negative value at high temperatures (not shown here), indicating the overestimation of bipolar thermal conductivity. Even though it is not likely to separate the bipolar thermal conductivity, the suppression of bipolar thermal conductivity can be qualitatively understood.

From the subtraction of electronic thermal conductivity $\kappa_{el}$, as presented in Figure 3c, we extract the lattice and bipolar thermal conductivity $\kappa_{lat}$ + $\kappa_{bi}$, as presented in Figure 3b. The lattice and bipolar thermal conductivity $\kappa_{lat}$ + $\kappa_{bi}$ values are significantly decreased for the MgO and VO_2_ dispersed bulk composites. MgO/VO_2_ BST composites’ electronic thermal conductivity is also significantly reduced by scatterings of carriers near the grain boundaries between matrix and oxide (MgO and VO_2_) micro-particles. From the EDX images, the MgO and VO_2_ exist randomly in a BST matrix, and some of MgO and VO_2_ were agglomerated. Moreover, the dispersion of MgO and VO_2_ particles scatters the phonon as well as carriers. We estimated the phonon mean free path $\lambda_{ph}$ by $\kappa_{lat}=\frac{1}{3}Cv_{s}\lambda_{ph}$, where the value of sound velocity $v_{s}$ was used 2070 m/s of the Bi_2_Te_3_ along the c-axis [30]. The calculated $\lambda_{ph}$ values are 7.28, 6.88, 6.65, 6.74, and 6.87 nm for BST, MgO 5% composite, MgO 10% composite, VO_2_ 5% composite, and VO_2_ 10% composite, respectively. It is not surprising that the phonon mean free path is shorter than the average grain size of extrinsic oxide particles because the phonon scattering is not solely from the grain boundary phonon scattering but also from various scattering mechanisms such as defects, dislocations, Umklapp process, and many imperfections. It implies that the extrinsic oxide phase distribution generates various scattering sources including defects, dislocations, and precipitations in matrix. Therefore, MgO and VO_2_ effectively scatter the heat-carrying phonon and charge carriers, resulting in reduced thermal conductivity κ. The suppression of lattice and bipolar thermal conductivity is beneficial to the decrease of total thermal conductivity.

**Figure 4:**
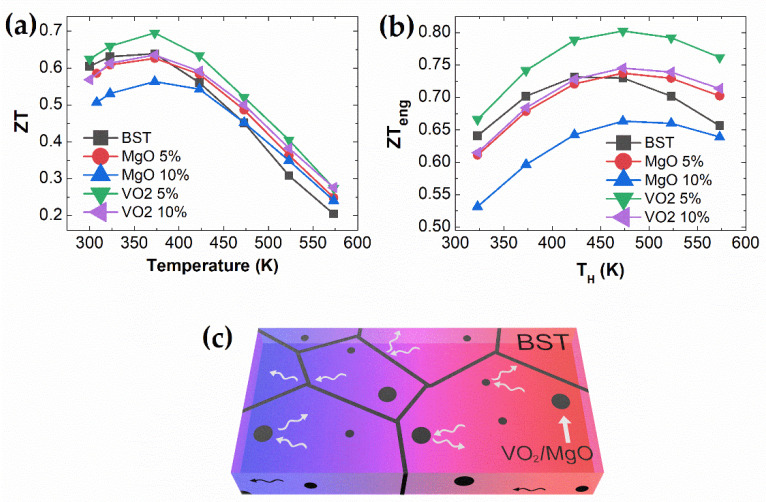
Temperature-dependent (**a**) ZT; (**b**) engineering dimensionless ZT with $T_{L}$ = 300 K; (**c**) schematic of phonon scatterings of MgO and VO_2_ BST composites.

Figure 4a,b depict temperature-dependent *ZT* and engineering *ZT_eng_* of MgO/VO_2_ BST composites. *ZT_eng_* is the dimensionless engineering *ZT* given by [31]

(12) $$ZT_{eng}=\frac{{\left(\int_{T_{c}}^{T_{h}}S\left(T\right)dT\right)}^{2}}{\int_{T_{c}}^{T_{h}}\rho \left(T\right)dT\int_{T_{c}}^{T_{h}}\kappa \left(T\right)dT}∆T=\frac{{\left(PF\right)}_{eng}}{\int_{T_{c}}^{T_{h}}\kappa \left(T\right)dT}∆T$$

where $T_{h}$, $T_{c}$, and ∆T are the hot/cold-side temperature and the temperature difference. The ZT value of VO_2_ 5 mol.% enhances the overall measured temperature range due to thermal conductivity reduction despite the decrease in power factor. In a high-temperature range, the ZT values of MgO 5 and VO_2_ 10 mol.% composites are increased compared to pristine BST, which is mainly caused by the suppression of the bipolar diffusion effect. It should be noted that the MgO and VO_2_ result in a beneficial effect in enhancing the thermoelectric performance by significantly reducing the thermal conductivity. Therefore, the ZT value of VO_2_ 5 mol.% is significantly enhanced over all temperatures. The ZT_eng_ value of MgO 5 and VO_2_ 10 mol.% composites are increased in the high-temperature range due to the reduction of thermal conductivity.

## 4. Conclusions

In summary, we investigated the thermoelectric properties of MgO/VO_2_ BST composites by extrinsic phase mixing of MgO and VO_2_ nanoparticles in the BST matrix. From the elemental mapping images from an HR-SEM, the MgO and VO_2_ nanoparticles are randomly agglomerated as a micrometer size scale within a matrix. MgO and VO_2_ distribution in the BST matrix effectively scatter phonons and electronic charge carriers, increasing electrical resistivity and considerably reducing electronic thermal conductivity. Because of an extrinsic phase mixing, the Hall carrier density is not sensitive to the MgO and VO_2_ concentrations, while there is a systematic decrease in Hall mobility. When we subtract the electronic thermal conductivity, the lattice and bipolar thermal conductivity of the MgO/VO_2_ BST composites are systematically decreased, implying the scattering of phonons and the asymmetric scattering of charge carriers. The reduction of thermal conductivity affects the enhancement of the ZT value over a wide temperature range, such as the BST/VO_2_ 5 mol.% composite. This research suggests that the extrinsic phase mixing of nanoparticles decreases lattice thermal conductivity and bipolar contribution of thermal conductivity, resulting in the enhancement of thermoelectric performance over a wide temperature range. 

## Figures and Tables

**Table 1:** The average grain size d, internal lattice strain ε, Hall carrier concentration $n_{H}$, Hall mobility $\mu_{H}$, and carrier effective mass $m^{*}$ of MgO and VO_2_ BST composites.

-	d (nm)	ε (10^−4^)	$n_{H}$ (10^19^ cm^−3^)	$\mu_{H}$ (cm^2^ V^−1^ s^−1^)	$m^{*}\left(m_{e}\right)$
BST	72	0.4568	3.45	189	0.8841
BST/MgO 5%	75	1.3825	3.17	161	0.8711
BST/MgO 10%	79	1.7254	2.94	146	0.8150
BST/VO_2_ 5%	75	1.2638	3.09	173	0.8540
BST/VO_2_ 10%	73	1.5063	3.23	168	0.8667

## Data Availability

The data presented in this study are available on request from the corresponding author.
